# Pharmaceutical Digital Marketing and Its Impact on Healthcare Physicians of Pakistan: A National Survey

**DOI:** 10.7759/cureus.2789

**Published:** 2018-06-12

**Authors:** Masood Jawaid, Syed J Ahmed

**Affiliations:** 1 Director Medical Affairs, Pharmevo (pvt) Ltd, Jinnah Sindh Medical University, Karachi, PAK; 2 Pharmevo (pvt) Ltd, Director Pharmacy Services, Alkhidmat Hospital, Karachi, PAK

**Keywords:** digital marketing, pharmaceutical marketing, pharmaceutical industry

## Abstract

Objective

Digital marketing is replacing traditional marketing strategies in the pharmaceutical industry. This study evaluated multiple aspects of the use of social media by the physicians of Pakistan, the current role of digital marketing in the pharmaceutical industry, and its impact on the change in clinical practice.

Methods

This cross-sectional study included physicians working in various clinical settings with at least five years of clinical experience. The participants were surveyed on social media tools used, knowledge of digital marketing tools, and the digital presence of themselves as physicians. We also assessed their knowledge of digital marketing by the pharmaceutical industry of Pakistan and its potential influence on changes in their clinical practice.

Results

Seven hundred eighteen physicians were included after taking informed consent. The mobile application WhatsApp (WhatsApp Inc., Menlo Park, CA) was the most frequently used application per week for medical-related purposes. Webinars/webcasts had the highest duration of use per week but were attended by only a few physicians, followed by mobile applications and informative health websites. The most frequently available digital marketing channels were found to be WhatsApp (29.94%), informative health websites (26.7%), and mobile applications (20.6%). Less frequently available tools were e-detailing (8.1%), webinars/webcasts (7.7%), tele-detailing (6.0%), self-directed web-detailing (5.2%), and marketing emails (4.2%). However, despite the limited use, webinars/webcasts had the strongest influence for changes in clinical practice (48%), followed by websites (42%), mobile applications (41%), WhatsApp (37%), and self-directed web-detailing (36%).

Conclusion

Despite limited use, the percentage of influence for clinical practice changes was highest for webinars/webcasts followed by websites, mobile applications, and WhatsApp. There is potential for increased use of digital promotion strategies from Pakistan’s pharmaceutical sector.

## Introduction

Digital media is an essential part of life. In the pharmaceutical industry, digital marketing is replacing traditional marketing strategies. This is primarily due to ease of interaction with end users, less time-consuming engagement, and great cost-effectiveness [1]. Moreover, the digital marketing has led to greater interest by physicians in use of social media and other digital tools [1-3]. Due to the wide use of social media, people can connect socially and share information with great ease [2]. This also affects how patients, physicians, and healthcare organizations interact [4]. For example, the World Health Organization (WHO) on the social network, Twitter (Twitter, San Francisco, CA), has more than 12,000 followers, and their feed is updated with diverse healthcare information. In the United Kingdom, more than half of the public uses Facebook (Facebook, Inc., Menlo Park, CA) to seek healthcare information and communicate about health issues. LinkedIn (LinkedIn Corp., Sunnyvale, CA) and Facebook are among the popular tools for healthcare interaction in European health organizations. The use of informative websites related to health is particularly prominent for inhabitants of the United States [3].

The provision of easy ways to access health care information for both patients and physicians justifies the global potential value of digital marketing [5]. However, the widespread availability of digital marketing by pharmaceutical firms has yet to be improved [6]. The limitation of this digital presence is primarily due to small numbers of reliable case studies, restrictions applied by the governing bodies, a lack of some drug literature, and less insight about digital approaches [1].

This study evaluated multiple aspects of the use of social media by physicians, as well as the current role of digital marketing and its impact on physicians. Input from physicians was vital to establish the benefits and level of use of digital tools in current health care practices in Pakistan. Moreover, the identification of gaps in awareness and the access and use of digital tools by physicians is essential to build future strategies.

## Materials and methods

This cross-sectional study included 718 physicians working at various clinical sites related to different specialties (convenience sampling) with at least five years of clinical experience. The data were collected through self-administered questionnaires after participants provided informed consent. After a short briefing, the participants were asked about the digital tools they currently use to assess medical information, their knowledge of digital marketing tools, the digital presence of the physicians, and the type and frequency of social media or digital media with social media elements used. For this study, social media is defined as websites and/or mobile applications where users can publish and share their own content and media with other users, including the ability to post comments, responses, and links to other media and/or websites. Their knowledge of digital marketing by the Pakistani pharmaceutical industry and the digital tools’ influence on changes in clinical practice were assessed. The data were analyzed in the IBM Statistical Package for Social Sciences (SPSS) Statistics for Windows, Version 23.0 (IBM Corp., Armonk, NY), and variables were summarized as frequencies and percentages.

## Results

A total of 718 physicians were included in the study after taking informed consent. The mean age of the physicians included was 41 ± 10 years. Five hundred thirty-eight participants (74.9%) were men and 180 (25.0%) were women. Most (31.1%) were from Lahore and 19.5% and 15.0% were from Karachi and Faisalabad, respectively. In addition, the study included doctors from Hyderabad (8.8%), Gujranwala (4.9%), and other cities (20.75%).

Most physicians (76.2%) reported using Facebook as their preferred social media platform, while WhatsApp and YouTube were used by 71.9% and 41.8% of physicians, respectively. Moreover, the use of Instagram (Facebook, Inc., Menlo Park, CA) (18.4%), Twitter (18.0%), and ResearchGate (ResearchGate GmbH, Berlin, Germany) (9.3%) was also observed. Only 16 physicians did not use any digital media with social elements. Half of the participants (50.1%) used social media during working hours for less than one hour, while 23.7% of them used social media for one to three hours (Table 1). Only 19.6% of them reported never using social media during working hours. When asked about digital doctor-patient interaction, 44.4% of doctors interacted with their patients on WhatsApp, while 28.6% and 13.1% of physicians preferred simple messaging service (SMS), a text messaging service component of most mobile device systems (SMS, McLean, VA), and Facebook, respectively.

**Table 1:** Social Media Services Used By Physicians and Duration of Use During Working Hours (N = 718) *Multiple social media used by single physician.

Social Media Use*	Frequency (%)
Facebook	547 (76.2)
WhatsApp	516 (71.9)
YouTube	300 (41.8)
Instagram	132 (18.4)
Twitter	129 (18.0)
ResearchGate or Academia	67 (9.3)
LinkedIn	64 (8.9)
Blogging Services (Word Press, Tumblr, etc.)	12 (1.7)
No social media usage	16 (2.2)
Social Media Use During Working Hours (per day)	
< 1 hours	360 (50.1)
1 to 3 hours	170 (23.7)
3 to 6 hours	37 (5.2)
> 6 hours	10 (1.4)
Never	141 (19.6)

For updating knowledge of medical information, 32.31% of physicians read medical websites. We found that 20.3% of participants used mobile applications, and some used WhatsApp/SMS (9.05%), webinars/webcasts (6.12%), e-detailing (8.21%), and tele-detailing (5.15%) (Table 2). Despite using WhatsApp for a brief duration (7 minutes/week), the frequency of use of WhatsApp per week was highest for medical-related uses (7.5 ± 12.6 times per week). The webinars/webcasts had the highest duration of use per week (31.67 ± 10.39 minutes), followed by mobile applications (26.5 ± 7.6 minutes), and informative health websites (10 ± 4.2 minutes) (Table 2).

**Table 2:** Preferred Digital Channels Use By Physicians When Assessing Medical Information n: number; SD: standard deviation

Digital Channel	Frequency (%)	Frequency of use per week	Duration of use (minutes/week)
		Mean ± SD	Mean ± SD
Website	232 (32.31)	4.68 ± 4.0	10 ± 4.2
Mobile applications	146 (20.3)	5.73 ± 3.7	26.51 ± 7.6
WhatsApp	65 (9.05)	7.55 ± 12.6	7.02 ± 1.6
e-Detailing	59 (8.21)	4.02 ± 2.4	3.29 ± 1.1
Webinars/Webcasts	44 (6.12)	0.2 ± 0.1	31.67 ± 10.3
Self-directed Web Detailing	16 (2.22)	3.88 ± 2.9	4.39 ± 1.2
Marketing Emails	33 (4.59)	4.06 ± 2.5	1.81 ± 0.6
Teledetailing (Phone calls)	37 (5.15)	4.84 ± 2.4	2.86 ± 1.0

When reporting their experiences with pharmaceutical industry digital marketing tools and the influence of those tools for change in clinical practice, the most frequently available digital channels were WhatsApp (29.94%), informative health websites (26.7%), and mobile applications (20.6%). Among the less frequently available tools were e-detailing (8.1%), webinars/webcasts (7.7%), tele-detailing (6.0%), self-directed web-detailing (5.2%), and marketing emails (4.2%). However, despite their limited use, webinars/webcasts had the highest reported percentage of influence for change in clinical practices (48%) as reported by the study physicians, followed by websites (42%), mobile applications (41%), WhatsApp (37%), and self-directed web-detailing (36%). In addition, marketing emails were found to be the least influential (8.0%) among all the available digital tools used by the pharmaceutical industry (Table 3).

**Table 3:** Available Digital Channels in Pakistan and Their Influence in Changing Clinical Practices of Physicians SMS: simple messaging service

Digital Channel	Frequency (%)	Influence for Change in Clinical Practice (%)
WhatsApp / SMS	215 (29.94)	37
Website	192 (26.7)	42
Mobile Apps	148 (20.6)	41
E-detailing	58 (8.1)	36
Webinars/Webcasts	55 (7.7)	48
Tele Detailing	43 (6.0)	34
Self-Directed Web Detailing	37 (5.2)	37
Marketing Emails	30 (4.2)	8

Only 31.7% of the total participants had a digital presence in a professional capacity on social media. One hundred ninety-two (26.7%) had a professional Facebook page, 149 (20.8%) relied on the webpage of their institute, and 77 (10%) relied on a personal website. Two hundred eighty-three (39.4%) received an inquiry from their patients in the digital space, and 336 (46.8%) were willing to consult their patient in a digital medium given the opportunity. Two hundred seventy-two (37.9%) said they usually discuss the use of social media during consultation with patients.

Most of the physicians surveyed in this study were aware of the digital marketing products of PharmEvo (PharmEvo (Pvt.) Ltd., Karachi, Sindh, Pakistan), such as digital continuing medical education (CME; 43.3%), eDrug index (34.4%), mobile applications (25.5%), Facebook page (16.6%), MedWeb (MedWeb, San Francisco, CA) (8.6%), web conferencing (7.4%), telemedicine (3.3%) and patient portals (2.6%) (Table 4). As perceived by 29.2% of the physicians, PharmEvo was rated as the most active pharmaceutical company for digital engagement followed by GlaxoSmithKline (GlaxoSmithKline, Sacramento, CA) (18.8%) and Getz Pharma (Getz Pharma (Pvt.) Ltd., Karachi, Pakistan) (11.42%). A majority of physicians in our study (73.0%) agreed that there is a strong need for two-hour workshops on digital marketing for them.

**Table 4:** Physician Knowledge of Digital Marketing Initiatives of PharmEvo CME: continuing medical education; FB: Facebook

Digital Marketing Products	Frequency (%)
Digital CME	311 (43.3)
eDrug Index	247 (34.4)
Mobile Applications	183 (25.5)
PharmEvo FB Page	119 (16.6)
Medweb	62 (8.6)
Web Conferencing	53 (7.4)
Telemedicine	24 (3.3)
Practice Management System	22 (3.1)
Customize Website for Doctors	22 (3.1)
Patient Portals	19 (2.6)

## Discussion

While most physicians in our study use digital tools with varying degrees of influence, digital marketing has room for improvement [1]. Pharmaceutical firms need to prioritize digital marketing strategies to beat the intensely competitive business environment [1]. Some of the suggested strategies include collaboration among the firms, more frequent conduction of case studies, launching mobile applications, building information technology (IT) and e-marketing teams, focusing on digitalization, and keeping physicians updated with the latest digital marketing tools.

Social media use permeates modern life, which includes the pharmaceutical marketing and the healthcare sectors. Nearly all (97.8%) study participants were active users of social media, which coincides with the consequences of the growth of social media in the United States described by Moorehead et al. [3]. The inclination of physicians towards digital tool use during working hours is understandable, given online information is easily accessible relatively quickly and, therefore, can save time. 

The prime targets of pharmaceutical marketing are physicians who are able to prescribe the products to the end users; the goal of pharmaceutical marketing is getting an authorized professional to advocate or recommend a product for end consumer use, which differs from other marketing efforts that focus directly to the end consumer. The pharmaceutical market is highly competitive, given the rarity of a physician’s time. To be effective, pharmaceutical marketing must be innovative and embrace new ways to engage their audiences, such as workshops, e-detailing, and digital sampling [7].

Social media and digital communication allow for an enhanced availability to patients. However, digital interactions between physicians and patients are limited [8]. Many of our study participants indicated a willingness to explore digital patient interactions.

The availability of digital tools influences the clinical practice of physicians and affects the doctor-patient relationship [9]. In the past, the common sources for increasing a physician’s medical knowledge were traditional libraries, paper textbooks, research journals, and CME sessions. More recently, however, digital knowledge platforms and their increased accessibility have revolutionized the search for medical information for physicians with decreasing amounts of time for such research [10]. Currently, digitally available tools are ubiquitous—not just for newer digital platforms and applications but for books and research journals as well, which are available in digital formats.

Traditional marketing techniques such as marketing emails and reminders have become less popular. This is primarily due to changes in the digital marketing industry that have created a higher rate of competition. Hence, most of the marketing emails land into the junk folder or remain unopened in the inbox. We found that webinars carried a greater influence for changes in clinical practice compared with other digital media. Webinars’ effectiveness for influencing change may be due to the focused nature of the discussion on a specific topic, the interactive question and answer sessions, and the one-hour time commitment. Physicians who take the time to attend a webinar session are already motivated to engage with that particular topic. The reasons for the effectiveness of webinars in influencing change is currently being explored [11]. It is important to note that information delivered in any industry-sponsored continuing medical education (CME) event (whether digital or more traditional) may carry some inherent sponsor bias in the information presented. Our study does not explore the impact of bias in CME or digital marketing, but future studies may be warranted to explore the intersection of sponsor bias and physicians’ clinical knowledge.

According to one study, pharmaceuticals companies do not allocate adequate resources to meet the current demands of digital marketing [12]. This is despite that, compared to other industries, pharmaceutical companies increased their spending on online marketing efforts 32.4% in 2013, the largest increase in resource spending for any area of marketing. Despite this increase, the pharmaceutical sector is still spending only a small percentage of their resources on digital marketing [13].

To the best of our knowledge, pharmaceutical companies in Pakistan spend very little of their marketing budgets on digital marketing efforts due to a lack of experience in digital marketing teams. Some Pakistani industries have recently assembled digital teams to focus on creating strategies and initiatives for digital marketing. This indicates digitalization in marketing will grow in time [12].

Digital marketing is the key to escalating the pharmaceutical industry [14]. Initially, digital marketing comprised only a few segments of pharmaceutical marketing, with a focus on over-the-counter medications. The trend has since escalated rapidly and included almost all prescription medications. Current focus has shifted to CME activities and e-detailing on product features [7]. The pharmaceutical sector is likely to extensively adopt the digital approach, which will serve as the backbone of the industry [1].

The number of in-person pharmaceutical representatives has dropped by approximately 20% to 30% [7]. Pharmaceutical firms have expanded their presence by utilizing social media, such as Facebook, Twitter, and other digital outlets, such as YouTube (YouTube, San Bruno, CA) to inform physicians on product details and related medical information. Online ordering provided by Quantum Pharma (Quantum Pharma Plc, Durham, United Kingdom) is an effort to keep ethics and regulations intact [7].

## Conclusions

Despite limited use, webinars/webcasts had the largest influence on changes to clinical practices. There is potential for increased usage of digital promotion strategies by Pakistan’s pharmaceutical sector.
